# Feeding in Gastric Ulcer

**Published:** 1902-03-08

**Authors:** 


### FEEDING IN GASTRIC ULCER.

In a striking, if somewhat popular, and *ad captandum* clinical lecture, recently delivered at St. Bartholomew's Hospital, Sir Lauder Brunton[1] described some of the reasons by which one is influenced in laying down a course of dietetic treatment for cases of gastric ulcer.

"Supposing," he said, "you have an ulcer on the back of your hand, how are you going to treat it? You know perfectly well you would not like it sandpapered three or four times a day, nor would you like to have it dressed with vinegar instead of with water. You would not like to have a poultice made of pickles, pepper and cayenne applied to it. You must treat a gastric ulcer in very much the same way; you must avoid irritating the ulcer by anything that will do harm to it, either mechanically or chemically."

As an example of sandpapering a gastric ulcer, he instanced the use of strawberry jam as a food, the achænia or seeds of the strawberry being absolutely indigestible, very hard, and pointed at one end. The kernels or ovules of seeds, such as almonds, nuts, and the like are also very hard so that anything in the shape of nuts or seeds or stones in fruits is to be avoided.

Then bones, as the bones of fish or broken splinters of bone such as are met with sometimes in curries and game pies, are very dangerous. Things also which act as a scrubbing brush are to be avoided, such as the cellulose found in the stalk of a cabbage, or in the skins of fruit, do a great deal of harm, and as in raisins and currants one cannot remove the stones and cannot remove the skins one must avoid the whole fruit. A patient with an ulcer of the intestine, as in typhoid fever, may be killed by a currant as surely as by a rifle bullet. Strings, which may do harm mechanically, are also met with in meat, and the greater digestibility of some kinds of meat depends on the lesser size and strength of the fibres it contains; those of beef being the coarsest and of chicken the finest.

Fibres and lumps in food may do harm chemically as well as mechanically. Anything which tends to plug the orifice of the pylorus tends to keep the food longer in the stomach, and the longer it stays there the more acid does it become, so that in cases of gastric ulcer, wheie the acidity is generally over the normal to begin with, there is a double reason for avoiding fibrous and lumpy food.

In gastric ulcer the fluid food *par excellence* is milk, but if milk is drank "a lot at once," it may coagulate and form a fibrous meat which may do great harm, so that one must give milk in small quantities, and combine it with lime water, or barley water, or soda water, or must peptonise it, by either of which the formation of hard indigestible curd may be prevented.

Hence, we begin by giving, in addition to nutrient enemata, only a tablespoonful of milk with a tablespoonful of lime water every two hours, gradually increasing the quantity as we find the patient is able to bear it without causing pain. As soon as it becomes desirable to give more solid food great care is to be taken that it is thoroughly masticated or mechanically broken up before it is swallowed, as it is only in this way that its digestion can be insured.

After milk has been employed for some days, the next stage is that of custards, which are very readily digestible, and so smooth that they can produce no mechanical injurious effect. Then from custards one may proceed to pounded fish, and from that to pounded meat, which if it is thoroughly broken up and mixed with fluid is really like milk. "You may make a very nice liquid food," says Sir Lauder Brunton, "of pounded chicken, which is very useful not only in cases of gastric ulcer, but in diseases of all kinds when patients will not take meat. You simply take the breast of a chicken, cut it into thin slices, then mince it up, put it into a mortar, grind it with a little chicken broth or else a little weak beef tea, until it is like a paste. Then mix it up with a little more chicken broth or beef tea until it is about the consistence of milk." Many a patient will drink this who will not swallow other meat preparations. Thus in treating gastric ulcer, first there is rest in bed and rectal feeding for a few days, then careful feeding by the mouth while the rectal feeding is continued, the mouth feeding consisting at first of a tablespoonful of milk and one of lime water every two hours, gradually diminishing the lime water and increasing the milk. Then custards, then pounded fish, then pounded meat, then chocolate (a very grateful change), and lastly the various forms of farinaceous foods, taking great care that in whatever form they are given they are so prepared as to be incapable of forming masses into which the pancreatic juice cannot gain access.

[1] Brit. Med. Jour., March 1.

